# Tracheal Erosion Secondary to Prolonged Endotracheal Intubation in a Patient With Bilateral Cerebellar Stroke: A Case Report

**DOI:** 10.7759/cureus.89105

**Published:** 2025-07-31

**Authors:** Mohamed Zuhail K Peediyakkal, Ashib Thurakkal, Saibu George, Dinesh Chengamaraju, Nevin Kannappilly, Saifil Sidhique, Abdulqadir J Nashwan

**Affiliations:** 1 Critical Care Medicine, Hamad Medical Corporation, Doha, QAT; 2 Nursing and Midwifery Research, Hamad Medical Corporation, Doha, QAT

**Keywords:** aspiration pneumonia, endotracheal intubation, prolonged ventilation, stroke, tracheal perforation, tracheostomy

## Abstract

Tracheal erosion is a rare but life-threatening complication of prolonged endotracheal intubation, especially in medically complex patients. We report a case of a 70-year-old male with multiple comorbidities, including bilateral cerebellar stroke, multi-infarct dementia, and heart failure with reduced ejection fraction, who developed tracheal erosion secondary to prolonged endotracheal intubation of 265 days. Computed tomography revealed that the endotracheal tube had pierced the anterior wall of the trachea. Due to extensive tracheal erosion and the patient’s poor surgical candidacy, tracheostomy was not feasible. The patient was managed conservatively under multidisciplinary care. This case highlights the diagnostic and management challenges of a tracheal injury in critically ill patients and reinforces the importance of early airway intervention, close tube monitoring, and individualized management strategies.

## Introduction

Tracheal erosion and perforation following endotracheal intubation is a rare but serious complication, with an incidence of less than 0.1% among intubated patients [1]. Risk factors include prolonged mechanical ventilation, elevated cuff pressures, improper tube placement, and pre-existing airway pathology [2]. Clinical manifestations can be subtle or masked, particularly in neurologically compromised individuals. Delayed diagnosis may result in serious consequences such as mediastinitis, pneumomediastinum, and sepsis [3].

Here, we describe a case of tracheal erosion in an elderly gentleman with a history of bilateral cerebellar stroke and multiple comorbidities, in whom tracheostomy was contraindicated due to anatomical erosion and clinical instability.

## Case presentation

A 70-year-old man was transferred to our tertiary care center from an overseas facility for ongoing management following a prolonged course (90 days) of mechanical ventilation and rehabilitation, post-bilateral cerebellar stroke. The initial cerebrovascular accident was attributed to vertebrobasilar artery occlusion, for which he had received intravenous thrombolysis. His prior medical history was significant for type 2 diabetes mellitus, long-standing hypertension, a prior ischemic stroke five years earlier with residual mild right-sided hemiparesis, and a diagnosis of multi-infarct dementia four years prior. He was also known to have heart failure with reduced ejection fraction.

Upon admission, the patient remained intubated, was hemodynamically stable but neurologically obtunded, with Glasgow Coma Scale fluctuating between 6 and 7 (E2VTM4), and showed minimal recovery in sensorium. He remained dependent on mechanical ventilation and required high-level nursing care.

**Figure 1:**
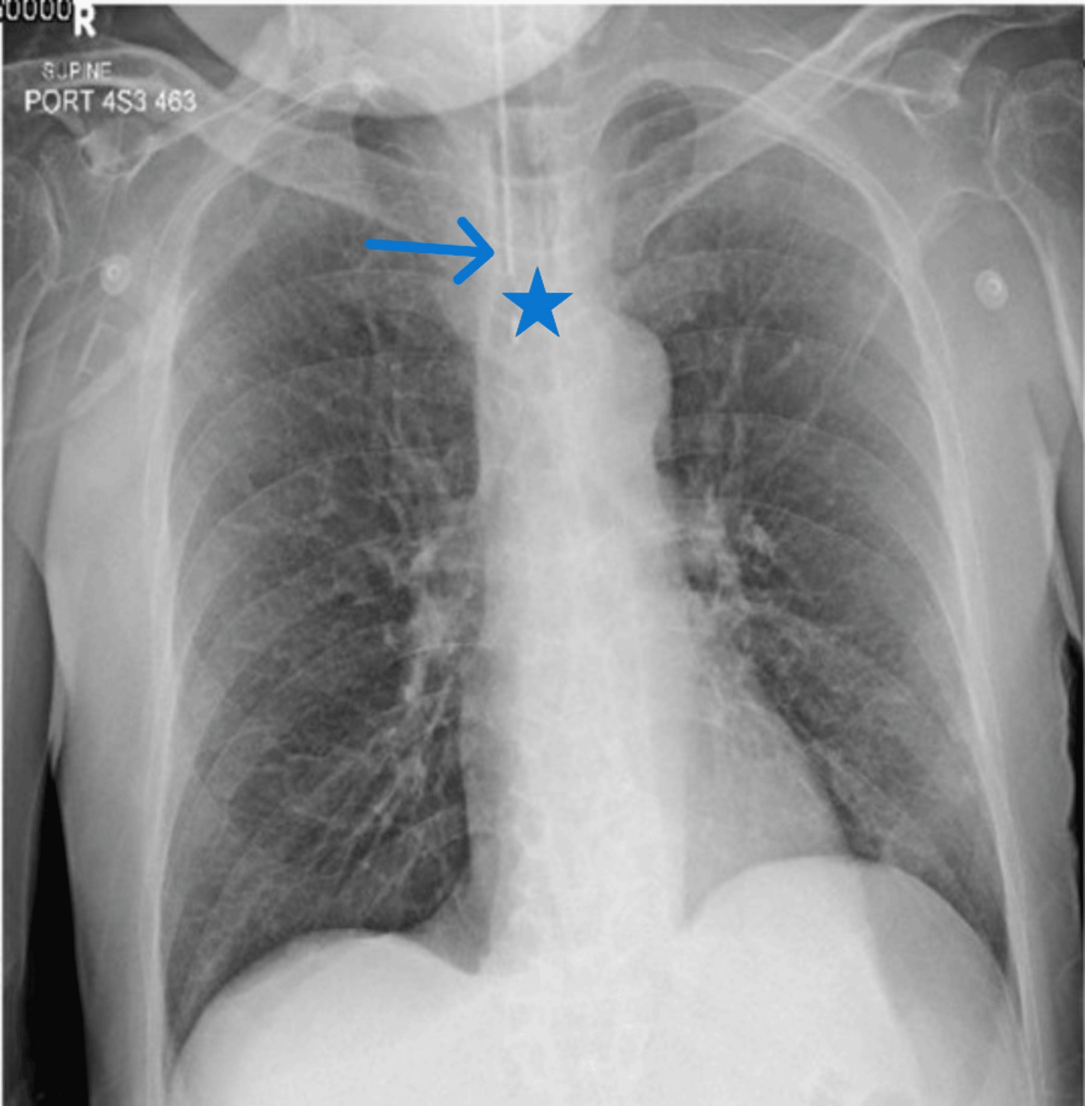
Chest X-ray demonstrating normal lung fields and proper endotracheal tube placement Anteroposterior chest X-ray showing clear lung fields bilaterally, indicating no evidence of consolidation, effusion, or pneumothorax. The endotracheal tube is visible and appears to be appropriately positioned within the trachea (arrow). The trachea is centrally located (star).

A chest X-ray performed on admission showed the endotracheal tube (ETT) apparently in situ within the trachea, with no immediate signs of gross displacement. Lung fields were clear, and there was no evidence of pneumomediastinum, subcutaneous emphysema, or pleural effusion (Figure 1). However, due to persistently high airway pressures, ventilation difficulties, and concerns raised during suctioning (presence of fresh blood), further imaging was warranted.

**Figure 2:**
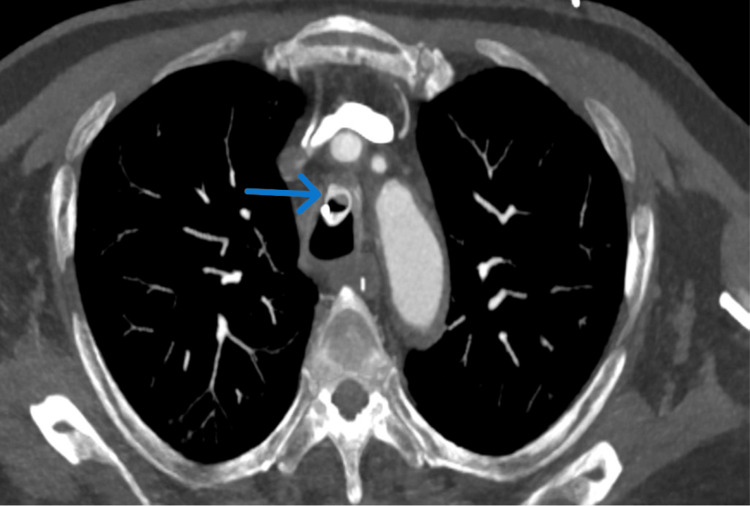
CT thorax showing tracheal erosion due to endotracheal tube malposition Axial CT image of the thorax demonstrates the tip of the endotracheal tube piercing the anterior wall of the trachea (arrow), confirming the presence of tracheal erosion. This finding highlights a rare but serious complication of prolonged or improperly positioned endotracheal intubation.

**Figure 3:**
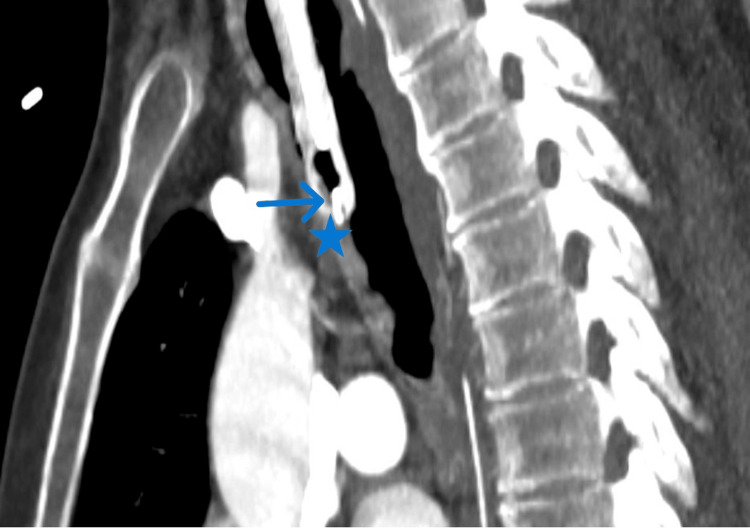
Sagittal reformatted MIP CT showing tracheal wall penetration by the endotracheal tube Sagittal reformatted maximum intensity projection (MIP) CT image demonstrates the tip of the endotracheal tube (ETT) (arrow) penetrating the anterior wall of the trachea (star), consistent with tracheal erosion.

A computed tomography (CT) scan of the thorax was subsequently performed to assess the airway anatomy and potential complications (Figure 2). The CT scan revealed a critical finding: the tip of the endotracheal tube had eroded through the anterior wall of the trachea, breaching the cartilaginous structure and protruding into the adjacent soft tissues of the anterior mediastinum. This was evident on axial and sagittal reformatted maximum intensity projection (MIP) images, which demonstrated a discontinuity in the anterior tracheal wall, confirming tracheal erosion (Figure 3).

These imaging findings confirmed a diagnosis of severe tracheal erosion, likely exacerbated by long-standing pressure from the ETT cuff, coupled with patient immobility, tissue fragility, and potentially unmonitored cuff pressures during prior hospitalization.

Following this diagnosis, urgent evaluations were undertaken by both otolaryngology and thoracic surgery teams. Given the extent of tracheal wall disruption, significant granulation tissue formation, and the patient's overall frailty and poor prognosis, surgical tracheostomy was deemed not feasible. It was felt that surgical intervention would further compromise the already fragile airway and carry a high risk of fatal airway collapse or hemorrhage.

The patient's clinical course was further complicated by the development of polymicrobial sepsis, likely secondary to aspiration and ventilator-associated pneumonia. Blood and urine cultures grew multiple organisms, including Klebsiella pneumoniae. Broad-spectrum intravenous antibiotics and antifungal agents were initiated in accordance with the sensitivity profiles, with regular input from infectious disease specialists.

Due to the poor surgical candidacy, a conservative, non-surgical management plan was adopted. The patient was transferred to a high-dependency unit (HDU) for continuous monitoring, with strict airway precautions, and regular surveillance imaging.

Despite the challenges, the patient remained under multidisciplinary care for over 8 months (265 days). He continued to require full ventilatory support and experienced repeated episodes of infection and delirium, managed with appropriate supportive therapies.

## Discussion

This case report presents a rare and complex clinical scenario of severe tracheal erosion secondary to prolonged endotracheal intubation in a 70-year-old gentleman with multiple significant comorbidities. The patient's underlying medical history, including multiple strokes, multi-infarct dementia, diabetes mellitus, and hypertension, significantly compounded the challenges associated with his prolonged mechanical ventilation and subsequent complications.

Endotracheal tube (ETT)-induced tracheal injuries, while a known complication of intubation, typically manifest as mucosal erosion, ulceration, or, in severe cases, tracheomalacia or tracheoesophageal fistula [4]. However, direct piercing of the anterior tracheal wall by the ETT tip, as observed in our patient, is an exceedingly rare and severe form of tracheal trauma. This highlights the importance of meticulous ETT placement and continuous monitoring of its position, especially in patients requiring prolonged intubation or those with altered anatomy or fragile tissues [5]. Factors such as patient movement, tube migration, cuff overinflation, or pre-existing tracheal conditions can predispose to such severe injuries.

The management of severe tracheal erosion poses significant challenges, particularly when conventional surgical interventions like tracheostomy are deemed unfeasible [6]. In our patient, the extensive nature of the tracheal erosion precluded tracheostomy, forcing a prolonged conservative management strategy. This decision, though challenging, underscores the need for individualized treatment plans based on the patient's overall clinical status, comorbidities, and the extent of the injury. The extended period of conservative management (265 days) in a high-dependency setting further illustrates the dedication to supportive care in complex cases where definitive surgical repair is not an immediate option.

The development of polymicrobial sepsis in this context is a grave complication, reflecting the patient's compromised immune status, prolonged hospitalization, and the potential for secondary infections originating from the compromised airway. This highlights the crucial importance of implementing rigorous infection control measures and administering prompt, broad-spectrum antibiotic therapy in patients with severe, chronic tracheal injuries.

This case serves as a poignant reminder for clinicians regarding the potential for severe and atypical ETT-related tracheal complications, particularly in vulnerable populations requiring prolonged mechanical ventilation. It highlights the diagnostic utility of chest imaging, such as CT thorax, in identifying unusual tracheal injuries [6]. Furthermore, it underscores the necessity of a multidisciplinary approach involving otolaryngology, thoracic surgery, critical care, and infectious disease specialists to navigate the complex diagnostic and therapeutic challenges presented by such severe tracheal erosion and its associated complications [7]. The long-term conservative management strategy, while demanding, demonstrated that supportive care can be maintained even in the face of profound anatomical injury when surgical options are limited.

This case highlights the crucial importance of early consideration for tracheostomy in patients anticipated to require prolonged intubation. It also highlights the need for routine monitoring of endotracheal tube positioning, cuff pressures, and early proper imaging in patients who show signs of respiratory compromise, persistent infection, or unexplained hemodynamic instability. Moreover, a multidisciplinary approach involving critical care, ENT, thoracic surgery, and infectious disease teams is crucial for making optimal decisions in complex cases like this [8].

While conservative management was the only viable option for our patient, the case serves as a cautionary example of the potential risks of delayed tracheostomy and the devastating impact of prolonged mechanical ventilation in medically fragile individuals. Clinicians should maintain a high index of suspicion for tracheal injury in similar settings and advocate for early preventive measures where feasible. Regarding the imaging modalities for diagnosing tracheal injuries, the trachea may appear normal on X-ray, even if erosion is present, unless there are indirect signs, such as air leakage or a significant mass effect, which are not always evident.

A CT scan, on the other hand, provides high-resolution, three-dimensional images of the chest, including the trachea and surrounding structures. It can directly visualize tracheal wall defects, thinning, or fistulas, and detect associated abnormalities such as mediastinal air, fluid collections, or tumor invasion [8]. CT's superior contrast and cross-sectional views make it ideal for identifying small or complex lesions that X-rays miss. In short, CT scans offer greater detail and sensitivity for soft tissue and airway structures, making them far more effective for diagnosing tracheal erosion than chest X-rays.

A review of recent literature reveals that tracheal erosion due to prolonged intubation remains a rare but serious complication, with only a limited number of documented cases in the past decade. Most of these cases involved patients with prolonged mechanical ventilation, high cuff pressures, or delayed tracheostomy, similar to the present case. Notably, previous reports, including the one by Zlotnik et al, have described rare but serious tracheal ruptures and erosions following prolonged intubation, often attributed to cuff overinflation, tissue fragility, and delayed recognition [9]. These cases underscore the need for vigilant airway monitoring in chronic, ventilated patients.

## Conclusions

Tracheal erosion is a rare but life-threatening complication of prolonged intubation, especially in patients with underlying frailty, neurological deficits, and delayed tracheostomy. This case underscores the need for proactive airway management, timely multidisciplinary evaluation, and individualized decision-making when tracheostomy is delayed. Clinicians should maintain a high index of suspicion and monitor for early signs of tracheal injury to prevent severe outcomes. Emerging technologies, such as continuous cuff pressure monitoring systems, pressure-relief endotracheal tubes, and real-time tracheal ultrasound, are being explored to mitigate risks. These tools, along with AI-assisted ventilator analytics, may offer early warning for airway complications and should be considered in high-risk patients in long-term critical care settings.
